# Prognostic value of lymph node staging systems in gastric cancer patients undergoing laparoscopic surgery: A case-series in Vietnam

**DOI:** 10.1016/j.ijscr.2025.111970

**Published:** 2025-09-21

**Authors:** Kien Quach Van, Tam Nguyen Thi Thanh

**Affiliations:** a Department of Digestive Surgery, Viet Duc University Hospital, Hanoi, Viet Nam; b Hanoi Medical University, Hanoi, Viet Nam

**Keywords:** Lymph node ratio, Log odds of positive nodes, Gastric cancer, Case series

## Abstract

**Introduction:**

Lymph node metastasis is the most important prognostic factor for gastric cancer (GC). While the pN staging system is widely used, it does not account for the total number of dissected lymph nodes, potentially leading to stage migration in patients with suboptimal lymphadenectomy. Alternative systems such as the lymph node ratio (LNR) and log odds of positive nodes (LODDS) may provide superior prognostic accuracy. Our aim is to compare the prognostic significance of the lymph node ratio (LNR), log odds of positive nodes (LODDS), and number of positive lymph nodes (pN).

**Methods:**

Eighty-six GC patients treated with curative laparoscopic surgery were retrospectively analyzed. Survival outcomes were assessed using Kaplan-Meier analysis and the log-rank test. Prognostic accuracy was evaluated using receiver operating characteristic (ROC) curves and the area under the curve (AUC) values.

**Results:**

All three lymph node classification systems, pN, LNR, and LODDS, were significant prognostic factors for survival in gastric cancer, (*p* = 0.007, 0.002, and 0.036, respectively) based on the log-rank test. Notably, in cases with fewer than 15 lymph nodes dissected, only the LNR system retained prognostic significance (*p* = 0.037), whereas both LNR and LODDS were effective in the ≥15 lymph node subgroup.

**Conclusions:**

LNR and LODDS can be recommended for evaluating lymph node metastasis in gastric cancer, particularly in patients with inadequate lymph node dissection. This is the first study in Vietnam to evaluate and support the integration of LNR and LODDS as complementary prognostic tools in gastric cancer staging.

## Highlights

• LNR (AUC = 0.685) and LODDS (AUC = 0.683) demonstrated better prognostic accuracy than pN (AUC = 0.677).
• LODDS may differentiate survival outcomes better than LNR in patients without lymph node metastasis (pN0).

## 1 Introduction

Gastric cancer (GC) is a common cancer and one of the leading causes of death worldwide. Currently, there have been significant advances in the treatment of GC. Research on the lymphatic system, the characteristics of lymph node metastasis in GC, the limits of gastric resection, lymph node dissection techniques, neoadjuvant and adjuvant chemotherapy and radiation therapy, and targeted therapy have all contributed to improving treatment quality, prognosis, and extending survival time for patients. However, surgery remains the primary treatment for GC, with D2 lymph node dissection being the standard procedure with the aim of curing advanced GC [1].

Regarding the number of lymph nodes dissected, the 8th edition of the AJCC recommends examining at least 16 lymph nodes for staging, but if possible, ≥30 lymph nodes should be checked for more accurate classification and prognosis [2]. The examination of ≥15 lymph nodes has improved the prognostic power of the AJCC 8th edition in patients with gastric cancer, excluding those with cardia gastric cancer [3]. Therefore, lymph node dissection of fewer than 15 nodes may be considered substandard and may reduce the quality of postoperative care. In the TNM staging system, pN is based on the number of metastatic lymph nodes without considering the number of lymph nodes dissected. As a result, this can lead to limitations in both tumor staging and survival prediction, especially in patients with suboptimal lymph node dissection [4]. Thus, the Lymph Node Ratio (LNR) system, which takes into account both the number of positive lymph nodes and the number of lymph nodes dissected, has shown increasing value. However, for cases with no positive lymph nodes, pN and LNR are similar. Therefore, the log odds of positive nodes (LODDS), which can assess both the number of positive and negative lymph nodes, is a new lymph node classification system that shows many advantages and holds promise for gastric cancer [5].

Thus far, no research in Vietnam have compared the prognostic significance of the pN, LNR, and LODDS systems after D2 lymph node dissection surgery in gastric cancer. Therefore, in this current study, we aim to compare the prognostic performance of the pN, LNR, and LODDS lymph node classification systems and identify the most suitable lymph node classification system to predict overall survival rates in this group of patients. This case series has been reported in line with the PROCESS guidelines [6].

## 2 Materials and methods

### 2.1 Location and time

Department of Digestive Surgery, xxx Hospital, from January 2016 to December 2023.

### 2.2 Patients

The study employed a census approach, including 86 gastric cancer patients who underwent laparoscopic subtotal gastrectomy and D2 lymphadenectomy. All surgical procedures were performed at a specialized center by surgeons experienced in gastrointestinal cancer surgery.

#### 2.2.1 Inclusion criteria

Patients diagnosed with gastric cancer through endoscopy and biopsy, with histopathological result confirming gastric carcinoma; Patients who underwent laparoscopic subtotal gastrectomy (R0 resection) and D2 lymph node dissection; Complete and available medical records for research purposes.

#### 2.2.2 Exclusion criteria

Recurrent gastric cancer, non-epithelial gastric cancers, or cancers from other sites that metastasized to the stomach; Patients with comorbidity (respiratory, cardiovascular, …) who were not eligible for laparoscopic surgery.

### 2.3 Research methods

Descriptive, retrospective research.

### 2.4 Research variables

Age, gender; Postoperative histopathological results (tumor location, invasion depth, lymph node metastasis, number of lymph nodes dissected); Survival time after surgery, analyzed using the Kaplan-Meier method.

### 2.5 Lymph-node staging system

Lymph nodes were classified according to the TNM (tumor-node-metastasis) system (7th edition) of UICC/AJCC, based on the number of metastatic lymph nodes: N0: No metastasis; N1: 1–2 positive lymph nodes; N2: 3–6 positive lymph nodes; N3: >6 positive lymph nodes.

Lymph Node Ratio (LNR): The ratio of positive lymph nodes to total number of lymph nodes dissected. LNR classification: LNR0: 0; 0.01 < LNR1 ≤ 0.1; 0.1 < LNR2 ≤ 0.25; LNR3 > 0.25 (based on the study by Chen Jian-hui and colleagues on 935 patients) [5].

Log odds of positive nodes (LODDS): The logarithm of the ratio between number of positive and negative lymph nodes harvested, calculated as:

$$\text{LODDS}=\mathrm{Log}\left(\frac{\text{Number of positive lymph nodes}+0,5}{\text{Number of negative lymph nodes}+0,5}\right)$$

LODDS classification: LODDS1 ≤ −1.5; −1.5 < LODDS2 ≤ −1.0; −1.0 < LODDS3 ≤ 0.0; LODDS4 > 0.0 (based on the study by Chen Jian-hui and colleagues on 935 patients) [5].

### 2.6 Statistical analysis: statistical analysis was performed using SPSS version 20.0

Overall survival rates were calculated using the life table method, and log-rank testing was used to assess statistical differences between groups. Survival curves were generated using the Kaplan-Meier method. The prognostic accuracy of different classification systems was compared using receiver operating characteristic (ROC) curve and the area under the curve (AUC). Statistical significance was considered at *P* < 0.05. No AI tools were used for data analysis, figure generation, or manuscript writing.

## 3 Results

### 3.1 Clinical characteristics and survival analysis

A total of 86 patients were enrolled, including 51 men and 35 women, with a mean age of 61.9 ± 11.6 years (range, 31–87 years). The average length of hospital stay was 7.91 ± 1.5 days. The in-hospital mortality rate was 0.0 %. Most patients had stable postoperative courses, with only 6 cases (7.0 %) experiencing complications. Specifically, five patients developed surgical site infections (Clavien-Dindo Grade I), one patient had early postoperative bowel obstruction, which was managed conservatively (Grade II). No severe complications (Grade III or higher) or deaths were recorded (Table 1).

**Table 1:** Clinical characteristics and survival rates of the research patient group.

Characteristics		N	Survival rate (%)	Mean survival time (months)	χ 2 value (log-rank test)	*P* value
Age	≤60	39	87.2	81.0 ± 3.3	0.733	0.392
	>60	47	83.0	76.8 ± 4.8		
Sex	Male	51	84.3	79.8 ± 3.8	0.000	0.990
	Female	35	85.7	77.3 ± 4.7		
Tumor size (cm)	≤3	48	93.8	87.0 ± 2.8	6.225	**0.013**
	>3	38	73.7	70.4 ± 4.8		
Degree of differentiation	Well/moderately	29	89.7	77.2 ± 6.4	0.041	0.839
	Poorly/ring cell	57	82.5	80.4 ± 3.3		
T stage (UICC 7)	Tis + T1	45	97.8	89.8 ± 2.1	18.029	**<0.001**
	T2	10	90.0	79.3 ± 7.2		
	T3	15	80.0	70.5 ± 5.6		
	T4	16	50.0	56.8 ± 5.5		
N stage (UICC 7)	N(−)	65	90.8	84.2 ± 3.0	7.256	**0.007**
	N(+)	21	66.7	62.5 ± 5.7		
LNR stage	LNR0	65	90.8	84.2 ± 3.0	15.167	**0.002**
	LNR1	4	75.0	78.0 ± 0.0		
	LNR2	9	77.8	71.8 ± 3.7		
	LNR3	8	50.0	44.0 ± 10.6		
LODDS stage	LODDS1	38	94.7	82.7 ± 2.9	8.575	**0.036**
	LODDS2	27	81.5	80.5 ± 4.9		
	LODDS3	18	77.8	67.3 ± 5.1		
	LODDS4	3	33.3	38.0 ± 17.0		
Number of LNs dissected	<15	29	82.8	82.5 ± 4.0	0.381	0.537
	≥15	57	86.0	79.8 ± 3.0		

**Fig. 1:**
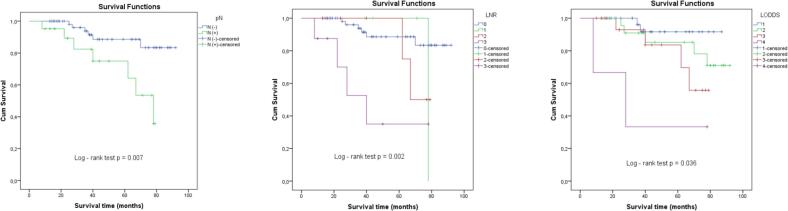
Kaplan-Meier analysis of overall survival for gastric cancer according to different lymph node staging systems: **a** patient survival curves according to the pN system, **b** patient survival curves according to the LNR system, and **c** patient survival curves according to the LODDS system.

The mean number of dissected lymph nodes was 18.1 ± 8.9 (range, 3–42). The mean number of positive lymph nodes was 1.3 ± 3.1 (range, 0–20). In 33.7 % of patients, lymph node dissection was inadequate. Our survival rate was 84.9 % with a mean survival of 79.8 ± 3.0 months (range, 8–92 months) (Fig. 1).

### 3.2 Comparison of prognostic performance among pN, LNR, and LODDS classification systems

To determine the most appropriate system to represent lymph node involvement for assessing overall survival in GC patients, we used the Akaike Information Criterion (AIC) and ROC curves to compare the prognostic performance among the systems. When applying the Cox regression model to the three classification systems, we found that the LNR system had the lowest AIC value, indicating better prognostic performance compared to pN and LODDS. Besides, regarding discriminatory ability, LNR also showed the highest AUC (0.685), followed closely by LODDS (0.683) and pN (0.677). Although the differences in AUC were small, all systems reached statistical significance (*p* < 0.05). These findings suggest that the three systems had relatively comparable prognostic performance, with both LNR and LODDS showing a modest advantage over pN. Further validation in larger cohorts is warranted (Table 2).

**Table 2:** Comparison of prognostic performance among different lymph node classification systems for gastric cancer.

Classification system	AIC	AUC (95 %CI)	P
pN (UICC 7)	99.159	0.677 (0.503–0.850)	0.039
LNR	95.333	0.685 (0.509–0.861)	0.034
LODDS	97.186	0.683 (0.509–857)	0.036

**Fig. 2:**
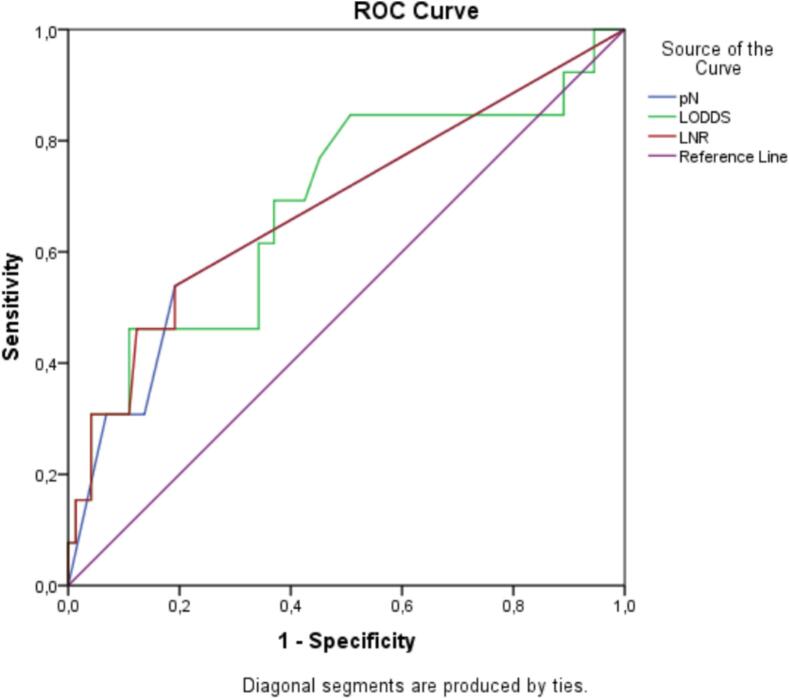
ROC curves for pN, LNR, and LODDS.

**Fig. 3:**
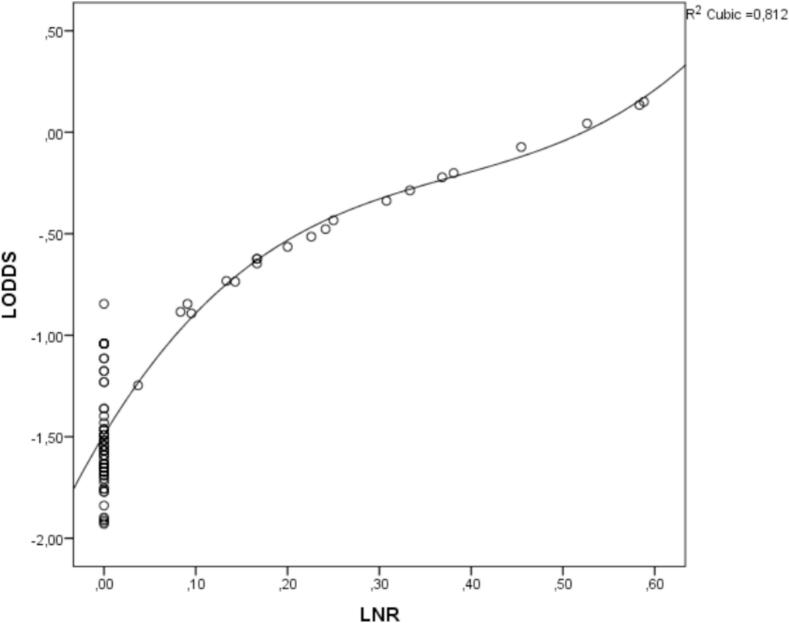
Scatter plot of the relationship between LODDS and LNR.

To compare LODDS and LNR, we generated a scatter plot of the relationship between LODDS and the lymph node metastasis rate. As shown in Fig. 2, the LODDS value increased with the lymph node metastasis rate, indicating a close relationship between LODDS and LNR. However, this correlation was not linear. When the lymph node metastasis rate was <0.2, the curve of LNR increased more slowly than that of LODDS, indicating that LODDS may be superior to LNR in predicting the long-term overall survival rate of the cases mentioned above. Furthermore, when the lymph node metastasis rate was 0, the LODDS score was not uniform, indicating that the LODDS system was likely to indicate different survival outcomes for patients with the same LNR stage, especially for cases with an LNR score of 0 (Fig. 3).

### 3.3 Correlation between the number of lymph nodes dissected, pN, LNR and LODDS

Pearson's test was performed to evaluate the correlation between the total number of lymph nodes retrieved and the pN, LNR and LODDS systems. There was a weak linear correlation between the number of lymph nodes dissected and LODDS (*r* = −0.287 and *P* = 0.007). However, there was no correlation between the number of lymph nodes dissected and pN, LNR (*r* = 0.168, *P* = 0.122 and *r* = 0.101, *P* = 0.354, respectively). Pearson's analysis results showed that LODDS had a strong linear correlation with pN and LNR (*r* = 0.869 and 0.873, *P* < 0.001 and < 0.001, respectively), while there was a close relationship between pN and LNR (*r* = 0.927, P < 0.001, respectively).

### 3.4 Evaluation of the prognostic value of different lymph node classification systems with different levels of lymph nodes removed

**Fig. 4:**
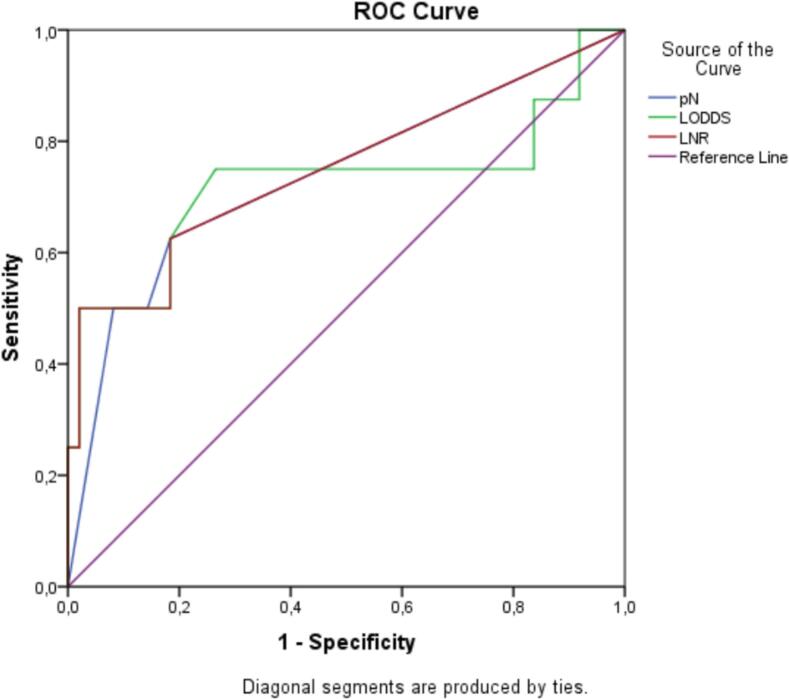
ROC curves for pN, LNR, and LODDS in patients with ≥15 dissected lymph nodes.

To evaluate the prognostic ability of pN, LNR and LODDS with different levels of lymph nodes removed, all patients were divided into two groups according to the number of lymph nodes removed: group 1 (<15, *n* = 29), group 2 (≥15, *n* = 57). As shown in Table 3, only LNR showed the ability to stratify prognosis between different subgroups, while pN and LODDS had prognostic value only when 15 or more lymph nodes were removed (Fig. 4).

**Table 3:** Evaluation of the prognostic value of different lymph node systems with different levels of lymph node dissection.

		N	Survival rate (%)	*χ*^2^ Value	*P* value
Number of LNs dissected < 15		29	82.8		
pN (UICC 7)	N (−)	22	86.4	1.180	0.227
	N (+)	7	71.4		
LNR	LNR0	22	86.4	8.467	**0.037**
	LNR1	2	100.0		
	LNR2	3	33.3		
	LNR3	2	100.0		
LODDS	LODDS1	0		1.356	0.508
	LODDS2	21	87.5		
	LODDS3	7	71.4		
	LODDS4	1	100.0		
Number of LNs dissected ≥ 15		57	86.0		
pN (UICC 7)	N (−)	43	93.0	6.048	**0.014**
	N (+)	14	64.3		
LNR	LNR0	43	93.0	23.409	**<0.001**
	LNR1	2	50.0		
	LNR2	6	100.0		
	LNR3	6	33.0		
LODDS	LODDS1	38	94.7	36.330	**<0.001**
	LODDS2	6	66.7		
	LODDS3	11	81.8		
	LODDS4	2	0.0

We further compared the prognostic performance between groups when more than 15 lymph nodes were dissected and found that the LNR system had the largest AUC value, followed by the LODDS and pN systems (Table 4).

**Table 4:** Comparison of prognostic value of classification systems when the number of lymph nodes removed is ≥15 nodes.

Classification system	AIC	AUC (95 %CI)	P
pN (UICC 7)	54.008	0.737 (0.523–0.951)	0.033
LNR	46.119	0.750 (0.531–0.969)	0.024
LODDS	49.211	0.724 (0.472–0.976)	0.043

## 4 Discussion

Lymph node metastasis remains a cornerstone in the prognostic evaluation of gastric cancer (GC), significantly influencing staging accuracy, treatment planning, and survival prediction. The present study provides a comparative analysis of three lymph node classification systems—pN, LNR, and LODDS—based on real-world surgical data from a high-volume Vietnamese center, aiming to identify a more reliable prognostic model, particularly in patients with limited lymphadenectomy.

### 4.1 Clinical relevance in Vietnamese surgical practice

Until now, the extent of lymph node metastasis has always been a decisive factor in prognosis and the indication for adjuvant treatment in gastric cancer. The pN lymph node classification system based on the number of metastatic lymph nodes according to the UICC classification (seventh edition) has been widely used. However, the pN classification system has the disadvantage that the accuracy of prognosis is affected by the number of dissected lymph nodes. The AJCC/UICC TNM classification (eighth edition) recommends dissection of at least 15 lymph nodes for reliable staging [2]. In Vietnam—as in many developing countries—multiple factors such as late-stage presentation, resource limitations, and anatomical variations may lead to fewer than 15 lymph nodes being dissected during gastrectomy. This poses a major challenge to accurate staging under the current TNM classification. Our study is the first Vietnamese analysis to show that LNR retains prognostic significance even when <15 lymph nodes are retrieved (*p* = 0.037), whereas pN and LODDS lose statistical power in this subgroup. These findings suggest that LNR may be a more practical and robust system for prognostication in everyday surgical practice, especially where ideal lymph node yield cannot always be guaranteed. There are still some reports showing that many cases do not have enough 15 lymph nodes dissected. According to our research, up to 33.7 % of patients had less than 15 lymph nodes dissected. Miao-zhen Qiu [7] researched on 730 patients and also showed that up to 21.6 % of patients had less than 15 lymph nodes dissected.

### 4.2 Early postoperative outcomes

Early postoperative results were highly favorable, with a 0.0 % mortality rate and a low complication rate (7.0 %), all of which were classified as Clavien-Dindo Grade I or II and managed with noninvasive treatment. These findings are consistent with several other studies conducted in Vietnam. For example, Nguyen Anh Tuan [8] reported only 4 minor complications (4 %) in 100 laparoscopic gastrectomy cases, all treated non-surgically. Similarly, Pham Van Nam [9] observed a 2.7 % complication rate in 74 patients, including one wound infection and one case of pneumonia, both managed with medical treatment.

### 4.3 The prognosis of survival after surgery

The average survival time of research is 79.8 ± 3.0 months. The shortest follow-up time is 8 months, the longest is 92 months. According to our research, the factors affecting the prognosis of survival included: tumor size, tumor invasion (T) and the degree of lymph node metastasis (according to pN, LNR and LODDS). The prognosis of survival according to pN, the survival rate of N (−) and N (+) is 90.8 %; 66.7 %, respectively; the survival time is 84.2 months; 62.5 months, respectively. According to Bui Trung Nghia [10], the survival rate after 5 years for N0, N1, N2, N3 is: 93.6 %; 54.4 %; 51.0 %; 0 %. Thus, the number of metastatic lymph nodes is an important prognostic factor in GC.

Regarding the prognosis of survival according to LNR: With LNR > 0.25, the survival rate and duration were 50.0 % and 40.0 months; significantly lower than LNR0 which was 90.8 % and 84.2 months. Alatengbaolide [11] researched on 710 GC patients also showed that the rate of lymph node metastasis is an independent prognostic factor independent of the number of dissected lymph nodes. When predicting the prognosis of gastric cancer, the staging system based on the rate of lymph node metastasis is more reliable than the system based on the number of lymph node metastasis regardless of the number of dissected lymph nodes. Muhammer Ergenç [12] researched on 333 GC patients who underwent gastrectomy for cancer showed that high LNR is significantly related to OS and can be used as an independent prognostic factor.

Regarding the survival prognosis according to LODDS: From LODDS 1 → 4, the survival rate and survival time decrease significantly, from LODDS1 with the survival rate and survival time of 94.7 % and 82.7 months, respectively, to LODDS4 with the survival rate and survival time of 33.3 % and 38.0 months, respectively. Villalabeitia Ateca [13] researched on 199 GC patients and showed that the 5-year survival rate with LODDS1, LODDS2, LODDS3 and LODDS4 was 72.4 %, 60 %, 29.1 % and 13.9 %, respectively. The higher the LODDS value, the lower the average survival rate and survival time.

### 4.4 Comparison of prognostic ability between pN, LNR and LODDS classification systems

According to our research, the LNR and LODDS classification systems with AUC values of 0.685 and 0.683 were slightly higher than that of pN at 0.677, indicating better prognostic ability, though the difference was not substantial. Overall, the three systems showed comparable prognostic accuracy, but LNR and LODDS may offer certain advantages in specific clinical situations—for example, LNR in cases of inadequate lymph node dissection and LODDS in pN0 patients. Jiang Zhu [14] meta-analyzed 27 researches and found that higher LNR was significantly associated with shorter OS in GC patients, even when subgroup analysis was performed using all the different factors. This research provides further evidence that LNR may be an independent prognostic indicator in GC patients and be used as a parameter in future classification systems. Chen Jian-hui [5] researched on 935 patients, Pengfei Gu [15] researched on 7620 patients and also concluded that LODDS is an independent prognostic factor superior to LNR and pN.

Regarding the correlation between LODDS and LNR, we used the Pearson test and generated a scatter plot of the relationship between LODDS and LNR. It can be seen that LODDS has a strong linear correlation with LNR (*r* = 0.873, *P* < 0.001), the LODDS value increases with the lymph node metastasis rate, indicating a close relationship between LODDS and LNR. However, when the lymph node metastasis rate is <0.2, the curve of LNR increases more slowly than that of LODDS, indicating that LODDS may be superior to LNR in predicting the long-term overall survival rate of the cases mentioned above. Moreover, when the lymph node metastasis rate is 0, the LODDS score is not uniform, indicating that the LODDS system is capable of indicating different survival outcomes for patients with the same LNR stage, especially for cases with an LNR score of 0.

The LNR staging system based on both the number of metastatic lymph nodes and the number of dissected lymph nodes can theoretically overcome the limitations of the pN staging system based on the number of metastatic lymph nodes only. In our research, when the number of dissected lymph nodes was less than 15, only the LNR staging system had prognostic significance (*p* = 0.037, log-rank test). However, the number of patients in this subgroup was relatively small, particularly within the LNR subcategories. Therefore, this should be considered a preliminary observation that requires validation in studies with larger sample sizes. Seong-Ho Kong [16] researched on 8949 patients with primary T1-T4a GC who underwent surgery and concluded that the relationship between the number of dissected lymph nodes and survival may be affected by stage migration in a center with a numerous gastric cancer. The LNR system may be a better choice to compensate for this effect, and the prognostic predictive value of this system increased with a higher number of dissected lymph nodes. However, the LNR system still has some limitations, including small sample size in some studies, retrospective design and non-validated cut-off values. Besides, there was no difference between the pN and LNR systems in patients with negative lymph nodes, suggesting that the LNR is not able to distinguish differences in survival in pN0 patients. In addition, the cut-off values of LNR reported in several researches were different [17,18]. Therefore, the LNR system cannot be considered as an alternative to the current pN staging.

In cases where 15 or more lymph nodes could be dissected, all three classification systems pN, LNR, and LODDS showed prognostic ability (with *p* values of 0.014; <0.001; <0.001, respectively; log-rank test). And when comparing the prognostic value of these three systems with each other, it can be seen that LNR is the most superior with an AUC value of 0.75, followed by pN with an AUC value of 0.737 and LODDS with an AUC value of 0.724.

### 4.5 Limitations of the study

This study has several important limitations that should be acknowledged. First, the small sample size reduces statistical power and limits the generalizability of the findings. Second, the retrospective design may introduce selection bias and limits control over confounding variables. Third, the cut-off points used for LNR and LODDS were adopted from previous studies and have not been validated in the Vietnamese patient population, which may affect the accuracy and clinical applicability of these metrics.

## 5 Conclusions

This study demonstrated that both LNR and LODDS may offer improved prognostic stratification compared to the traditional pN staging system in gastric cancer patients undergoing curative gastrectomy, particularly in cases with inadequate lymph node dissection. Among these, LNR appears to be a potentially useful indicator in patients with fewer than 15 lymph nodes retrieved, a scenario frequently encountered in Vietnamese surgical practice. The LODDS system seems to be significant in the absence of lymph node metastasis. However, given the small sample size and retrospective nature of this study, these findings should be interpreted with caution and require validation in larger, multicenter studies using standardized cutoffs tailored to local populations.

## Consent

Written informed consent was obtained from the patient for publication and any accompanying images. A copy of the written consent is available for review by the Editor-in-Chief of this journal on request.

## Ethical approval

The study was retrospectively and the ethical approval by our research committee, Viet Duc University Hospital, Hanoi, Vietnam was exempted.

## Guarantor

Kien Quach Van, M.D, Ph.D.

## Research registration number

No needed because it is not the first case conducted in human.

## Provenance and peer review

Not commissioned, externally peer reviewed.

## Funding

None.

## Author contribution

Kien QV: Main surgeon, methodology, data analysis, revise the manuscript

Tam NTT: Collect the data, write the manuscript

## Conflict of interest statement

None.

## Data Availability

Upon reasonable request.
